# Clozapine rechallenge or continuation despite neutropenia or agranulocytosis using colony-stimulating factor: A systematic review

**DOI:** 10.1177/02698811231154111

**Published:** 2023-02-16

**Authors:** Olivier Corbeil, Laurent Béchard, Émilien Fournier, Maude Plante, Marc-André Thivierge, Charles-Émile Lafrenière, Maxime Huot-Lavoie, Sébastien Brodeur, Anne-Marie Essiambre, Marc-André Roy, Marie-France Demers

**Affiliations:** 1 Faculty of Pharmacy, Université Laval, Québec City, QC, Canada; 2 Institut Universitaire en Santé Mentale de Québec, Québec City, QC, Canada; 3 CERVO Brain Research Centre, Québec City, QC, Canada; 4 Centre Hospitalier Universitaire de Sherbrooke, Sherbrooke, QC, Canada; 5 Faculty of Medicine, Université Laval, Québec City, QC, Canada; 6 School of Psychology, Faculty of Social Sciences, Université Laval, Québec City, QC, Canada

**Keywords:** Clozapine, human colony-stimulating factor, neutropenia, agranulocytosis, rechallenge

## Abstract

**Objectives:**

Rechallenge/continuation of clozapine in association with colony-stimulating factors (CSFs) following neutropenia/agranulocytosis has been reported, but many questions remain unanswered about efficacy and safety. This systematic review aims to assess the efficacy and safety of rechallenging/continuing clozapine in patients following neutropenia/agranulocytosis using CSFs.

**Methods:**

MEDLINE, Embase, PsycInfo, and Web of Science databases were searched from inception date to July 31, 2022. Articles screening and data extraction were realized independently by two reviewers, according to Preferred Reporting Items for Systematic reviews and Meta-Analyses (PRISMA) 2020 systematic review guidance. Included articles had to report on at least one case where clozapine was rechallenged/continued using CSFs despite previous neutropenia/agranulocytosis.

**Results:**

Eight hundred forty articles were retrieved; 34 articles met the inclusion criteria, totaling 59 individual cases. Clozapine was successfully rechallenged/continued in 76% of patients for an average follow-up period of 1.9 years. There was a trend toward better efficacy reported in case reports/series, compared with consecutive case series (overall success rates of 84% and 60%, respectively, *p*-value = 0.065). Two administration strategies were identified, “as-needed” and prophylactic, both yielding similar success rates (81% and 80%, respectively). Only mild and transient adverse events were documented.

**Conclusions:**

Although limited by the relatively small number of published cases, factors such as time of onset to first neutropenia and severity of the episode did not seem to impact the outcome of a subsequent clozapine rechallenge using CSFs. While the efficacy of this strategy remains to be further adequately evaluated in more rigorous study designs, its long-term innocuity warrants considering its use more proactively in the management of clozapine hematological adverse events as to maintain this treatment for as many individuals as possible.

## Introduction

Clozapine (CLZ) is the most efficacious antipsychotic used in treatment-resistant schizophrenia (TRS), including in the early stage of treatment, which affects nearly one-third of all patients with schizophrenia (Leclerc et al., 2021; Meltzer, 1997; Williams et al., 2017). Although it often represents their only opportunity for recovery and reduces suicidality, CLZ use in schizophrenia is as low as 4% in Canada, 2.5%‒5.5% in the United States, and 4.3%‒12.8% in the United Kingdom (Bachmann et al., 2017; Latimer et al., 2013; Siskind et al., 2016; Stroup et al., 2014; Whiskey et al., 2021). These low rates are largely due to the potential occurrence of neutropenia/agranulocytosis, which require a strict hematological surveillance (Gee et al., 2014). Indeed, neutropenia/agranulocytosis, which can be life-threatening, occur in 3.8% and 0.4%‒0.9% of CLZ users, respectively (Li et al., 2020; Myles et al., 2018). Of note, these higher rates of neutropenia, compared to other antipsychotics, could also be explained in part by surveillance bias resulting from stricter hematological monitoring as well as undiagnosed cases of benign ethnic neutropenia (BEN) (Taylor et al., 2022). Neutropenia/agranulocytosis induced by CLZ mostly occur during the first 6–12 months of treatment (Alvir et al., 1993; Schulte, 2006). Other risks factors for developing neutropenia while receiving CLZ include being younger, Afro-American, having low baseline absolute neutrophil count (ANC) values and using drugs also associated with neutropenia (Lally and Flanagan, 2016).

In most countries, ANC values below 1.5 × 10^9^ cells/L during CLZ treatment warrant its discontinuation, but this threshold can be lowered for individuals with BEN. In the United States, since 2015, this threshold has been lowered to 1.0 × 10^9^ cells/L, which can be further reduced to 0.5 × 10^9^ cells/L for people with BEN (Bastiampillai et al., 2016). Unsurprisingly, discontinuation of CLZ following neutropenia can have tremendous consequences as a significant proportion of patients will quickly relapse from their TRS, which will in turn represent a major step back in their quest for recovery (Meltzer et al., 1996). For this reason, strategies allowing CLZ rechallenge or treatment pursuit despite neutropenia are a subject of growing interest, even though it remains on an off-label basis except in the United States (Bastiampillai et al., 2016). So far, both lithium and granulocyte colony-stimulating factors (G-CSFs), or less frequently granulocyte-macrophage colony-stimulating factors (GM-CSFs), have been used off-label in this context.

Lithium has been used to allow CLZ rechallenge for several decades now, perhaps because psychiatrists were already familiar with this drug. Its use is associated with acute and chronic leukocytosis, an effect which could be mediated by a mobilization of peripheral neutrophils and an increased neutrophils production in the bone marrow (Focosi et al., 2009). In a recent review though, despite this strategy being successful in 87% of the published cases (82/94), existing concerns regarding a potential masking effect of lithium could not be eliminated as its discontinuation seemed to be quickly followed by blood dyscrasias (Boazak et al., 2018). Moreover, lithium use isn’t without risks, as it also has its deal of adverse effects, and long-term use can lead to nephropathy, insipidus diabetes, and thyroid disorders (Grandjean and Aubry, 2009). As for colony-stimulating factor (CSF), these recombinant hematopoietic growth factors stimulate the production of neutrophils and are mainly used in oncology for patients receiving myelosuppressive chemotherapy (Kuderer et al., 2007). Their use for patients treated with CLZ was first described for the treatment of agranulocytosis in order to reduce its duration and prevent its associated complications (Lally et al., 2017b). It was only later that these agents were used in adjunct with CLZ to allow its rechallenge/continuation following neutropenia. Despite two reviews published in 2017, both highlighting the potential usefulness of this strategy, several questions remain unanswered before such approach can be more widely used in clinical practice (Lally et al., 2017a; Myles et al., 2017). On one hand, only case reports/series, as well as one retrospective cohort study, had been published at the time these two reviews were undertaken. As these types of studies are prone to important publication bias, the reported success rates of this strategy are less likely to reflect their real clinical efficacy. On the other hand, while two different CSF administration strategies have been identified, that is, “as-required” and “prophylactic,” the former was only reported in nine cases (Lally et al., 2017a; Myles et al., 2017). Thus, the finding that the “as-required” use of CSF might be associated with better efficacy than the “prophylactic” approach still needs to be better documented (Lally et al., 2017a; Myles et al., 2017). Addressing these elements was therefore deemed relevant in order to better support clinical practice and provide people dealing with TRS the best opportunity to a fulfilling life.

## Objectives

The main objective of this systematic review was to evaluate the efficacy and safety of CLZ rechallenge/continuation using CSF following a previous episode of neutropenia or agranulocytosis. Secondary objectives were to assess the efficacy of different strategies of CSF administration as well as to identify potential predicting factors of either successful or unsuccessful CLZ rechallenge/continuation.

## Methods

This systematic review was conducted in accordance with the Preferred Reporting Items for Systematic reviews and Meta-Analyses (PRISMA) 2020 guidance (Page et al., 2021). The online application *Covidence Systematic Review Software* (Veritas Health Innovation, Melbourne, VIC, Australia), specifically designed to facilitate conductance of systematic review by multiple reviewers, was used for the title/abstract and full-text screenings. The protocol was not registered.

### Search strategy

Online searches were conducted in MEDLINE, EMBASE, Web of Science, and PsycINFO electronic databases. EF designed the search strategy, which was independently reviewed by two information specialists of Laval University (see Supplemental Material). The date ranges searched were from databases’ inception date until July 31, 2022. There were no language restrictions; for articles written in languages other than English, French or Spanish, translation services were used. Study methodologies included in this review were case reports or series, retrospective or prospective cohort studies, cross-sectional studies, case-control studies or randomized-controlled trials. Included articles’ references lists were manually searched to retrieve other relevant studies. EF performed the electronic searches. EF and OC reviewed the abstracts of all identified studies to identify papers reporting on CLZ, neutropenia/agranulocytosis, and CSF. Potentially relevant papers were independently reviewed in full-text by E.F. and O.C. In instances where insufficient information was provided to assess eligibility, authors were directly contacted to obtain further details. In one case, the journal’s publisher provided an article’s missing supplemental table. Disagreements between the two reviewers about studies’ eligibility were resolved via consensus through discussion; arbitration was not required.

### Inclusion and exclusion criteria

Included articles had to report on at least one patient for whom CLZ had been reintroduced or maintained in combination with the use of CSF following a neutropenia or an agranulocytosis developed while they were on CLZ. Reports of patients for whom a rechallenge strategy with CSF was established, but never administered, were excluded. Since the use of CSF in oncologic settings has already been extensively documented, articles that reported on patients who developed blood dyscrasia while undergoing treatment for cancer were excluded.

### Definitions

CLZ rechallenge was defined as resuming the use of CLZ after the drug being previously discontinued for more than 7 days following an episode of neutropenia or agranulocytosis. CLZ treatment continuation was defined as maintaining CLZ despite an episode of neutropenia or agranulocytosis. In both situations, two different CSF administration strategies were considered, based on previous reviews (Lally et al., 2017a; Myles et al., 2017).

First, the use of CSF on a regular basis for a fixed duration, irrespective of ANC values, was classified as a “prophylactic strategy.” Second, the use of CSF only when required according to ANC values was classified as an “as-needed strategy.” Neutropenia was defined as ANC values below 1.5–2.0 × 10^9^ cells/L, while an ANC less than 0.5 × 10^9^ cells/L was considered as agranulocytosis. As for CSF, all available formulations of either G-CSF or GM-CSF, including bio-similar agents, and administered subcutaneously, intramuscularly, or intravenously, were included in this review.

### Outcomes

For the evaluation of efficacy outcomes, the CSF administration strategy was deemed successful if CLZ could be maintained at the end of follow-up, whether neutropenia or agranulocytosis episodes reoccurred during that span or not. Conversely, the CSF administration strategy was considered unsuccessful if CLZ had to be discontinued due to blood dyscrasia or its consequential complications (e.g., infections). Discontinuation of CLZ for reasons other than hematological did not constitute failures. For the purpose of statistical analyses, two groups of patients were formed on the basis of efficacy outcome, namely those for whom CLZ could be continued at the end of follow-up and those for whom the treatment had to be discontinued as mentioned above. As for the safety of CSF, any adverse events reported from its use were collected.

### Data extraction

Data were independently extracted from each included article by two reviewers, OC and EF, and any discrepancies were reviewed by a third reviewer (LB). Patients’ characteristics were collected (i.e., age, sex, ethnic origin, and main psychiatric diagnosis) and all neutropenia episodes occurring during a patient’s follow-up were documented. For each episode, the following variables were collected: delay of onset following CLZ initiation or rechallenge (or delay since last neutropenia episode in cases of CLZ treatment continuation); ANC nadir; CLZ dose at onset of neutropenia; CLZ treatment outcome (continuation or discontinuation); duration of time between neutropenia and CLZ rechallenge, when applicable; use of CSF concomitantly with CLZ; CSF administration strategy (“prophylactic” or “as-needed”); CSF formulation, route of administration, dosage, administration schedule, and ANC thresholds for the “as-needed” administration; concomitant lithium use; length of follow-up (i.e., duration of time between last neutropenia and end of follow-up or CLZ discontinuation); ongoing CLZ use at the end of follow-up; and CSF adverse events. When available, contributing factors or alternative causes of neutropenia/agranulocytosis were collected in order to assess CLZ accountability.

### Statistical analyses

Patients were divided into two groups based on their outcomes and comparisons were made using student’s two-sample *t*-test or chi-squared test. Analyses were carried out using SPSS^®^ version 28 (IBM Analytics, Armonk, NY, USA).

## Results

**Figure 1.**
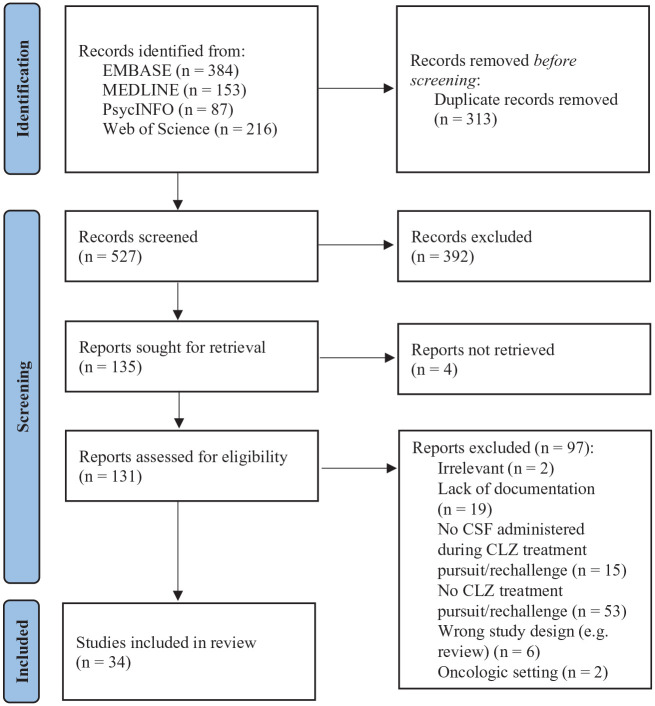
Flow chart of reviewed articles. Source: Adapted from Page et al. (2021). CLZ: clozapine; CSF: colony-stimulating factor.

A total of 840 articles were retrieved from electronic searches, from which 313 duplicates were removed as displayed in Figure 1. Review of title and abstract identified 135 potentially relevant articles that were reviewed in their entirety. From these, a total of 34 publications were included in this review; 4 of those reported on cases that were described more than once and were thus excluded from the analysis (Béchard et al., 2020, 2022; Demers et al., 2016, 2020). One case was also reported in two different articles and was therefore only included once in this review (Meyer et al., 2015; Spencer et al., 2012). As detailed in Table 1, 27 articles were case reports or non-consecutive series (total *n* = 38 cases) (Broughton et al., 2012; Comacchio et al., 2016; Conus et al., 2001; Elmore et al., 2016; Fabre-Bernal et al., 2019; Foster et al., 2017; Freeman et al., 2016; Friedman et al., 2017; Gopalakrishnan et al., 2013; Gugger and Caley, 2007; Hagg et al., 2003; Hazewinkel et al., 2013; Huguet et al., 2013; Joffe et al., 2009; Karst and Lister, 2018; Khan et al., 2013; Majczenko and Stewart, 2008; Martina et al., 2019; Mathewson and Lindenmayer, 2007; Morrow et al., 2020; O’Neill et al., 2021; Rajagopal et al., 2007; Ruiz-Doblado et al., 2020; Sengupta and Harvey, 2010; Shuman et al., 2021; Spencer et al., 2012; Sperner-Unterweger et al., 1998), while there were only 2 consecutive case series (*n* = 15 cases) (Béchard et al., 2021; Silva et al., 2021) and 1 retrospective cohort study (*n* = 6 cases) (Meyer et al., 2015), for a total of 59 individual cases.

**Table 1.** CSF success rate according to study methodology.

Study methodology	Number of studies	Number of cases	CSF strategy success rate			*p* Value
			As-needed	Prophylactic	Overall	
			*n*/*N* (%)	*n*/*N* (%)	*n*/*N* (%)	
Case reports/series	27	38^a^	13/14 (93)	15/18 (83)	32/38 (84)	0.065
Consecutive case series/retrospective cohort study	3	21^b^	8/12 (67)	5/7 (71)	13/21 (62)	

CSF: colony-stimulating factor.

a CSF strategy not specified for six cases.

b CSF strategy not specified for two cases.

### Patients’ characteristics

Among the 59 cases for whom the information was provided, 75% were men with a mean age of 41.6 years old (±14.7, range 17–71) and most were Caucasians (79%). CLZ was most frequently first prescribed for TRS (*n* = 57) but was also used for bipolar disorder (*n* = 1) and psychosis associated with Parkinson’s disease (*n* = 1). Following CLZ initiation, a first neutropenia episode occurred after an average of 4.6 years (±6.9, median = 0.5, range 0.01–25) and 60% arose during the first year of treatment. At the time of occurrence of this neutropenia, the mean CLZ daily dose was 357 mg (±162, range 25–600). During that first neutropenia episode, the average ANC nadir was 0.98 × 10^9^ cells/L (±0.40, median = 1.10, range 0.07–1.60), but the exact value was not specified in 23 cases. There were six cases of agranulocytosis (17%) and 12 patients had ANC values between 0.5 and 1.0 × 10^9^ cells/L (33%). Following this first episode, and after either CLZ rechallenge or pursuit, most patients had one or more subsequent neutropenia episodes (85%). These occurred quicker than for the initial episode in 70% of the cases and the ANC nadir was also lower during the second episode for 74% of the patients.

### Efficacy outcomes

Over an average follow-up period of 1.9 years (±2.2, median = 1.0, range 0.04–10.0) following CLZ rechallenge/continuation, CLZ was successfully maintained for 45 out of all 59 patients (76%). As displayed in Table 2, there were no statistically significant differences between patients for whom CLZ was maintained and those for whom it was discontinued. As for CSF strategies, success rates for the “as-needed” and “prophylactic” approaches were similar, when specified (81% and 80%, respectively). There was a trend toward higher success rates in case reports and non-consecutive case series, compared with consecutive case series and the only retrospective cohort study (overall success rates of 84% and 60%, respectively, *p*-value = 0.065). CSF was used in combination with CLZ after an average of 1.9 neutropenia episodes (±1.1, median = 2.0, range 1–7) and patients further experienced 1.1 episodes following CSF initiation (±1.8, median = 1.0, range 0–9). When reported, G-CSF were more frequently used than GM-CSF, as the latter was used for only two patients, and filgrastim was the most prescribed agent (*n* = 34), followed by lenograstim (*n* = 4) and pegfilgrastim (*n* = 2). Filgrastim was mainly administered subcutaneously (*n* = 14), but also intramuscularly (*n* = 2), and doses ranged from 150 (*n* = 2) to 480 μg (*n* = 5); the 300-μg dose was most frequently preferred (*n* = 25). When the “as-needed” strategy was employed, the most common thresholds prompting CSF administration were ANC values below 1.0 (*n* = 5) and 1.5 × 10^9^ cells/L (*n* = 3). As for the “prophylactic” strategy, CSF was generally administered once to three times weekly.

**Table 2.** Comparison of patients based on CLZ treatment outcome.

Variables	CLZ discontinued at follow-up (*n* = 14)	CLZ continued at follow-up (*n* = 45)	*p* Value
	*n* (%)	*n* (%)	
Age, mean ± SD, y	46.4 ± 14.2	39.4 ± 14.7	0.200
Gender			
Male	8 (80)	28 (74)	1.000
Female	2 (20)	10 (26)	
Ethnicity			
Caucasian	7 (78)	20 (80)	1.000
Non-Caucasian	2 (22)	5 (20)	
Main diagnosis			
Schizophrenia	13 (93)	44 (98)	0.421
Other	1 (7)	1 (2)	
Time to first neutropenia, mean ± SD, y	3.0 ± 4.4	5.3 ± 7.7	0.334
⩽1 year, *n* (%)	7 (58)	18 (60)	1.000
>1 year, *n* (%)	5 (42)	12 (40)	
ANC nadir at first neutropenia, mean ± SD, ×10^9^ cells/L	1.12 ± 0.28	0.90 ± 0.43	0.109
⩽1.00 × 10^9^ cells/L	5 (46)	13 (52)	0.717
>1.00 × 10^9^ cells/L	6 (54)	12 (48)	
CLZ dose at first neutropenia, mean ± SD, mg/d	404 ± 142	333 ± 172	0.334
Time to second neutropenia, mean ± SD, y	0.7 ± 1.2	0.3 ± 0.5	0.357
Delay shorter than first neutropenia	3 (27)	6 (32)	1.000
Delay longer or equal to first neutropenia	8 (73)	13 (68)	
ANC nadir at second neutropenia, mean ± SD, ×10^9^ cells/L	0.87 ± 0.47	0.71 ± 0.56	0.502
ANC nadir lower than at first neutropenia	3 (43)	3 (19)	0.318
ANC nadir higher or equal to first neutropenia	4 (57)	13 (81)	
Number of neutropenia episodes prior to CSF initiation, mean ± SD	1.9 ± 0.9	2.0 ± 1.2	0.933
Number of neutropenia episodes following CSF initiation, mean ± SD	1.7 ± 1.9	0.9 ± 1.8	0.234
CSF administration strategy			
As-needed	5 (50)	21 (51)	1.000
Prophylactic	5 (50)	20 (49)	
Concomitant lithium use	6 (75)	9 (41)	0.215
Follow-up, mean ± SD, y	2.6 ± 3.3	1.7 ± 1.8	0.501

ANC: absolute neutrophil count; CSF: colony-stimulating factor; d: day; L: litre; mg: milligram; SD: standard deviation; y: year.

### Safety outcomes

There were no deaths related to hematological complications nor CSF administration. Adverse events associated to CSF use were only documented for 34 cases out of 59. There were no adverse events noted for most of them (28/34) and the remaining patients experienced only minor events, including rebound leukocytosis (*n* = 2), mild euphoria (*n* = 1), flu-like symptoms (*n* = 1), short-lived back pain (*n* = 1), and splenomegaly, not clinically significant (*n* = 1).

## Discussion

In this review, the use of CSF to allow CLZ rechallenge/continuation despite neutropenia was found to be successful for 76% of the 59 included cases. This result is in line with two previous reviews, both published in 2017, in which success rates of 75 and 76% were observed based on 32 and 30 patients, respectively (Lally et al., 2017a; Myles et al., 2017). However, these latter findings were based solely on case reports or series, as well as one retrospective cohort study (Meyer et al., 2015), while two consecutive case series have since been published and included in the present review (Béchard et al., 2021; Silva et al., 2021). As could be expected given the reporting bias inherent to case reports/non-consecutive case series, the success rate of 84% reported in these studies was higher than the rate of 60% observed in consecutive case series/retrospective cohort studies. While this lower success rate found in more rigorous study designs may be more reflective of clinical reality, it is nonetheless considerable, especially given the innocuity of CSF. Indeed, only minor and transient adverse events associated with the use of CSF were reported over a mean follow-up period of 1.9 years. Still, in the absence of randomized controlled trials assessing this strategy, it is not possible to conclude on whether or not using CSF is associated with better outcomes than not using it, since patients for whom CSF is used probably differ from those for whom it is not. Indeed, it is likely that CSF use is guided by the degree of certainty about the causality of CLZ in a given case. For instance, CSF was used following a second neutropenia episode for 50% of all patients included in this review, meaning that for these patients, the first rechallenge without CSF had failed; such recurrence increases the likelihood of a causal role of CLZ. Unfortunately, it is impossible to compare the degree of certainty about the causality of the role of CLZ as the information provided in most published cases was not sufficient to properly assess this probability.

Based on available data, it is not yet possible to establish the superiority of any specific CSF administration protocol. Myles et al. (2017) concluded, respectively, that 70% of patients who received prophylactic CSF were still on CLZ at the end of follow-up, compared with 100% of patients who received it on an “as-needed” basis. Meanwhile, Lally et al. (2017a) also reported on a 70% effectiveness rate for CSF prophylactic use, compared with 89% for the “as-needed” strategy. However, these high success rates relied only on a small number of cases, that is, seven and nine cases, respectively. Such a clear-cut distinction between these two approaches was not evidenced in this present review, as CSF prophylactic use was successful in 80% of the cases (*n* = 20), compared with a success rate of 81% for the “as-needed” strategy (*n* = 21). This difference is partly driven by a recent consecutive case series in which there were four unsuccessful cases associated with the “as-needed” CSF administration scheme (Béchard et al., 2021). A less ambiguous finding from this present review is that G-CSFs are preferred over GM-CSFs and that the short-acting formulation of filgrastim has been most widely used in this particular setting. Considering that many patients do not require prolonged CSF use, it would seem most adequate to administer single doses of filgrastim 300 μg whenever ANC values drop below a prespecified threshold, such as 1.0 × 10^9^ cells/L, at least during the first few weeks following CLZ rechallenge. This would prevent unnecessary utilization of CSF, since human recombinant CSFs remain costly despite the availability of less expensive biosimilar versions, while not jeopardizing patients’ safety. Indeed, should neutropenia reoccur following rechallenge, it should be caught earlier on provided that blood monitoring is done weekly for at least the first 6 months. In the event of recurrent neutropenia requiring multiple CSF doses, a prophylactic strategy could be initiated with once to thrice weekly administrations of filgrastim. In any case, hematologists should be actively involved whenever a CLZ rechallenge is undertaken following blood dyscrasia.

As for potential predicting factors of either successful or unsuccessful CLZ rechallenge or pursuit, no characteristics were found to differentiate individuals for whom CLZ could be maintained or not at follow-up. Noteworthy, although not statistically significant, patients for whom CLZ rechallenge was successful had lower ANC nadir at first neutropenia episode than those for whom rechallenge was unsuccessful. Furthermore, agranulocytosis was experienced by 6 patients at first blood dyscrasia event, among whom 5 were nevertheless successfully rechallenged with CLZ. These findings suggest that rechallenging a patient who developed CLZ-induced agranulocytosis should not necessarily constitute an absolute contraindication, under strict follow-up conditions.

Results of this review need to be interpreted while taking into account some limitations. First, as discussed previously, non-consecutive case reports/series are prone to publication bias. Although two of the 30 included articles were consecutive case series, representing together 27% (16/59) of all included cases (Béchard et al., 2021; Silva et al., 2021), case reports/non-consecutive case series still account for a majority of the included articles. In order to further limit the impact of publication bias and to get a clearer picture of the efficacy and safety of CSF use to allow CLZ rechallenge/continuation, prospective cohort studies with larger samples of consecutive patients would be particularly relevant. Second, even though the number of included cases is roughly twice those included in Lally et al.’s (2017a) and Myles et al.’s (2017) reviews, the relatively small sample of 59 cases still limited the capacity to detect potential predictive factors to either CLZ rechallenge/continuation success or failure with the adjunction of CSF. The paucity of data provided in numerous of the included records further contributed to this issue. Third, one important element undocumented in almost all cases was the assessment of causality between CLZ treatment and occurrence of neutropenia/agranulocytosis. Indeed, other causes for neutropenia were only rarely explicitly explored, such as the use of other medications associated with hematological toxicity (e.g., anticonvulsants such as carbamazepine and valproate, antibiotics such as trimethoprim-sulfamethoxazole and penicillins) as well as conditions such as infections, metabolic disorders, and BEN (Curtis, 2017). Therefore, a favorable outcome of CLZ rechallenge/continuation while using CSF might result partly from a lesser risk of recurrence of neutropenia due to a dubious involvement of CLZ in the initial one. Additionally, while first episodes of neutropenia typically occur within the first year of CLZ treatment in more than 90% of all patients, the fact that this proportion was only 60% of all cases included in this review could suggest that for a relatively large number of patients, blood dyscrasias may not have been related to CLZ (Alvir et al., 1993; Schulte, 2006). Finally, this present review couldn’t explore the clinical outcome of patients for whom CLZ could successfully be rechallenged/continued nor for those for whom CLZ had to be discontinued since objective measurement of the disease’s severity was detailed in only one article (Béchard et al., 2021).

## Conclusion

While statistical power remains limited by the relatively small number of published cases, factors such as time of onset to first neutropenia and severity of the episode do not necessarily impact the outcome of a subsequent CLZ rechallenge using CSF. Thus, although the efficacy of CSF use to allow successful CLZ rechallenge remains to be adequately evaluated using more robust study designs, its long-term innocuity warrants considering its use more proactively in the management of CLZ hematological adverse events as to maintain this treatment for as many individuals as possible. Although there are insufficient data pointing toward any difference in terms of efficacy and safety between administering CSF on a prophylactic basis or “as-needed” only, the latter could improve accessibility to this costly medication. Further research is essential to further identify which patients would be better suited for this strategy. Hopefully, the development of new treatment strategies that ensure the safety of CLZ treatment, even in the occurrence of serious adverse events, will promote its widespread use and offer patients the best chance of recovery.

## Supplemental Material

sj-xlsx-1-jop-10.1177_02698811231154111 – Supplemental material for Clozapine rechallenge or continuation despite neutropenia or agranulocytosis using colony-stimulating factor: A systematic review Click here for additional data file. Supplemental material, sj-xlsx-1-jop-10.1177_02698811231154111 for Clozapine rechallenge or continuation despite neutropenia or agranulocytosis using colony-stimulating factor: A systematic review by Olivier Corbeil, Laurent Béchard, Émilien Fournier, Maude Plante, Marc-André Thivierge, Charles-Émile Lafrenière, Maxime Huot-Lavoie, Sébastien Brodeur, Anne-Marie Essiambre, Marc-André Roy and Marie-France Demers in Journal of Psychopharmacology
